# Synergistic Effects of 2-Deoxyglucose and Diclofenac Sodium on Breast Cancer Cells: A Comparative Evaluation of MDA-231 and MCF7 Cells

**DOI:** 10.3390/ijms26104894

**Published:** 2025-05-20

**Authors:** Geofrey Ouma Maloba, Tom Were, Erick Barasa, Nasreldeen Mohamed, Arshi Arshi, Ferenc Gallyas

**Affiliations:** 1 Department of Biochemistry and Medical Chemistry, University of Pécs Medical School, 7624 Pécs, Hungary; maloba.geofrey.ke@gmail.com (G.O.M.); karshom.nasreldeen@pte.hu (N.M.); arshi@pte.hu (A.A.); 2 Department of Pathology, Masinde Muliro University of Science and Technology, Kakamega 190-50100, Kenya; twere@mmust.ac.ke (T.W.); barasaerick256@gmail.com (E.B.)

**Keywords:** 2-deoxyglucose, diclofenac sodium, MDA-231, MCF7

## Abstract

Resistance of breast cancers to chemotherapy remains a global challenge to date. Drug combination studies between anti-cancer agents are increasingly becoming therapeutic strategies, geared towards alleviating breast cancers. Previously, 2-deoxyglucose has been shown to target and interrupt glycolysis. Available evidence also suggests that diclofenac, which was originally designed as a pain reliever, could inhibit the proliferation of breast cancer cells. However, the reverse Warburg effect and other metabolic reprogramming mechanisms in breast cancers limit the pharmacological application of both 2-deoxyglucose and diclofenac as mono-therapeutic agents. The present study explores the additive anti-cancer effects of 2-deoxyglucose and diclofenac sodium on breast cancer cells. In this study, MDA-231 and MCF7 cells were treated with 2-deoxyglucose and diclofenac sodium in single and combination doses before being evaluated for viability, cell growth, reactive oxygen species, apoptotic and necrotic phases, and migration abilities. Additionally, immunoblotting of pro-apoptotic proteins, Caspase-3 and Caspase-9, and a hypoxia-inducible factor-1 alpha, was also performed. The results showed that combination treatments of the cells with the drugs exhibited additive anti-cancer effects by limiting proliferation, enhancing cytotoxic reactive oxygen species generation, enhancing apoptosis and necrosis, limiting colony formation and expansion of cells, and inhibiting cell migration. The degrees of cytotoxicity of combined treatments were almost similar in both cell lines, although with minimal differences. Put together, these results reveal the novel synergistic effects of 2-deoxyglucose and diclofenac sodium on breast cancer cells, hence potentially elevating their pharmacological profile in the overall breast cancer therapy.

## 1. Introduction

Breast cancer (BC) remains a global health challenge with approximately 1.7 million diagnosed cases and over 0.5 million mortalities reported annually [1]. Despite substantial advances in therapeutic developments, high costs, BC reversions, resistance, and often undesirable side effects are associated with most commonly available therapies [2]. Triple-negative breast cancer (TNBC), such as MDA-231 cell types, are known to be more invasive and resistant to therapies than their counterparts, HER2+ and MCF7-hormone receptor-positive subtypes [3,4,5]. Nevertheless, both types have no single conclusive therapy to circumvent reversions and negative side effects. Further, the costs of chemotherapies for both cancer subtypes remain overly high. As such, there is still a need to continuously explore economically viable, effective, and safe therapeutic strategies to combat BCs in general. Drug combination studies between anti-cancer agents are therefore increasingly becoming a therapeutic strategy, geared toward this end [6]. Since diclofenac sodium salt (2-(2,6-dichlorophenyl)-amino benzo-acetic acid) and 2-deoxyglucose are a promising pair of effective anti-cancer agents [7,8,9] with potentially subtle side effects, exploring their overall synergistic anti-cancer effect on BC cells undoubtedly provides a novel frontier of cost-effective and easily available therapeutic options for BCs, with low-grade side effects. Due to mechanistic aerobic glycolysis (Warburg effect), malignant BCs, like other solid tumors, preferentially utilize glucose as the primary source of metabolic fuel to enhance their plasticity and proliferation [10,11,12]. 2-Deoxyglucose (2-DG) has recently been used to target glycolytic pathways in cancer cells, thereby revealing its therapeutic potential [7,8,13,14]. 2-DG is a non-metabolizable glucose analogue, which functionally inhibits phosphoglucose isomerase enzyme activity during glycolysis, hence blocking the eventual synthesis of glucose-6-phosphate (G-6-P) [15]. As previously documented, cancer cells have intrinsically high affinity for glucose substrate during metabolism. As such, the cells invariably incorporate 2-DG into their glucose transporters (GLUTs) as the alternative glucose molecules whenever glucose is limited [16]. Once inside the cells, 2-DG inhibits G-6-P synthesis [15] and eventually shuts down glycolysis. This mechanism is thought to hamper cell growth [7], thus elevating 2-DG as a potential therapeutic agent for BCs. While the targeting of glucose metabolism with 2-DG cannot be over-emphasized, its use as monotherapy in BC management remains unapproved and hence limited. Nevertheless, increasing evidence suggests that 2-DG could be effectively used to synergize other BC cytotoxic agents during chemotherapy. For instance, 2-DG has been shown to enhance the efficacy of mitochondria-targeted drugs (MTDs), such as Mito-CP and Mito-Q in BC chemotherapy, as well as lower mitochondrial bioenergetics and colony formation, especially when used together with a mitochondrial bioenergetic inhibitor, MDIVI-1, in TNBC cells, hence enhancing the cellular toxicity of BCs in general [14,17]. In addition, the co-treatment of BC with 2-DG and polydatin has been shown to inhibit ROS/PI3K/Akt/HIF-1α/HK signaling axis, thus leading to an overall substantial tumor regression [18]. Further, studies have also suggested that the combined treatment of TNBCs with 2-DG and metformin could limit cell survival, induce immune regulation of PD-L1 expression, and cause anoikis [5,19,20,21]. Additional reports have also documented that the dual treatment of BC with 2-DG and metformin enhances the pharmacological effects of sodium iodide symporter-mediated, targeted radioiodine. On the other hand, diclofenac sodium is a non-steroidal anti-inflammatory drug (NSAID) that has overtly been used as an anti-inflammatory, antipyretic, and analgesic agent over the years [22]. Diclofenac is administered by several routes, such as oral or rectal repositories, intramuscular or intravenous injections, and topical applications through the skin or as eye drops [22,23]. Diclofenac sodium exerts its effects by, among other mechanisms, the inhibition of cyclooxygenases 1 and 2 (COX-1, and COX-2), with particular bias on the selective inhibition of prostaglandin-endoperoxides synthase 2 (PGES-2), or also called (COX-2) [24]. Other studies have also revealed that diclofenac sodium limits the lipoxygenase pathway, reduces leukotriene biosynthesis, and inhibits phospholipase A2 (PLA 2) during inflammatory suppression [25,26,27]. Additional evidence also shows that diclofenac impairs glycolysis in the glucose transporter 1 (GLUT1) and hexokinase (HK) protein expression, particularly in BCs, and subtly inhibits peroxisome proliferator-activated receptor gamma (PPAR-γ), thereby blocking both cellular proliferation and survival in both TNBCs and non-TNBCs [28]. Put together, diclofenac sodium and 2-DG are potentially an incredible pair of economical and viable therapeutic regiments for BCs, with reduced chances of accompanying side effects. However, both agents have not individually been approved for use as monotherapies, and hence, their application in BC pharmacotherapy remains limited. In addition, their additive anti-cancer effects on individual TNBC (MDA-231) and hormone receptor-positive (MCF7) BC cells when used in combination have remained elusive. Accordingly, by evaluating the synergistic effects of 2-DG and diclofenac on MDA-231 and MCF7 cells on viability, apoptosis/necrosis, ROS production, cell migration, and colony formation, the present study shows that the two drugs, when used together in their pharmacological doses, exhibited enhanced anti-cancer effects on the two cell lines, thereby revealing their synergistic therapeutic value in breast cancers.

## 2. Results

### 2.1. Cell Viability

**Figure 1:**
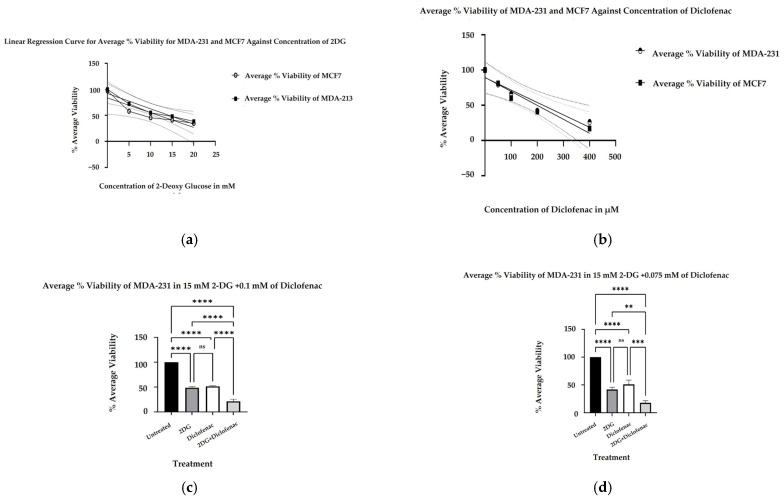
(MTT cytotoxicity assay) MDA-231 and MCF7 cells were separately plated for overnight incubation before treating with increasing concentrations of 2-DG (0 mM, 5 mM, 10 mM, 15 mM, and 20 mM) and diclofenac sodium concentrations (0 µM, 50 µM, 100 µM, 200 µM, and 400 µM) for 72 h. Percentage viabilities of cells were then determined from the optical density values of the purple formazan product formed following an MTT assay. For each treatment in each cell line, experiments were repeated three times (*n* = 3), with each experiment run in triplicate. (**a**,**b**) are linear regression curves showing the cytotoxic effects of different concentrations of both 2-DG and diclofenac sodium on the cell lines. (**c**,**d**) show viabilities of both cell lines in different treatments. All graphs were drawn to show mean and ±SEM. *p* ≤ 0.05 was considered significant in both assays. ns indicates no significant differences between the groups. Significantly different groups, ** = *p* < 0.01, *** = *p* < 0.001, **** = *p* < 0.00001, respectively.

Following the treatment of cell cultures with increasing concentrations of 2-DG and diclofenac sodium and subsequent MTT assay, optical density (OD) readings of the purple formazan product were measured as indicative of mitochondrial oxidoreductase activity, which is directly proportional to the number of viable cells remaining. The cell viabilities were normalized with blank (DMSO) and calculated as percentages in both cell lines. A linear regression model of the results of three independent experiments (*n* = 3) in both cell lines revealed significant linear relationships between the percentage viabilities and concentrations of single treatments of 2-DG and diclofenac sodium (Figure 1a,b) in MDA-231 and MCF7 cells, respectively, with significant reduction in percentage viabilities with the increase in drug concentrations. Thus, in MDA-231, *p* < 0.0138 and *p* < 0.0092 for 2-DG and diclofenac sodium, respectively, while in MCF7, *p* < 0.0002 for both 2-DG and diclofenac sodium. From the initial experiments and linear regression models, the IG50 doses for both drugs in both cell lines were then separately determined. The IG50 doses for 2-DG and diclofenac sodium were 15 mM and 0.1 mM, respectively, in MDA-231 cells and 15 mM and 0.075 mM, respectively, in MCF7 cells, in line with previous studies [14,17,18,19,20,21,29,30,31]. Following the Bliss independence model [32,33], when combination treatments of three independent experiments (*n* = 3) involving the calculated IG50 doses were made and results were compared with untreated and single treatment samples of both 2-DG and diclofenac sodium, we determined that the effect of the drugs on both cells lines was as follows: 2-DG_15mM_/diclofenac sodium_0.1mM_ > 2-DG_15mM_ and 2-DG_15mM_/diclofenac sodium_0.1mM_ > diclofenac sodium_0.1mM_ for MDA-231 cells, while 2-DG_15mM_/diclofenac sodium_0.075mM_ > 2-DG_15mM_ and 2-DG_15mM_/diclofenac sodium_0.075mM_ > diclofenac sodium_0.075mM_ for MCF7 cells. The combined effect of the drugs was thus higher than that of single treatments, indicative of synergy/addictiveness. Multiple comparisons of mean viabilities in one-way ANOVA Turkey post hoc showed that, in MDA-231 cells, cell viabilities in combination treatments reduced significantly when compared with both untreated and 2-DG-only- and diclofenac sodium-only-treated samples (*p* < 0.001) in both groups (Figure 1c). Similarly, in MCF7, cell viabilities also reduced significantly when combination treatment samples were compared with both untreated and 2-DG-treated samples (*p* < 0.001) in both cases when compared with diclofenac sodium-treated samples (Figure 1d). Results revealed, however, that there was no significant change in parentage viabilities of cells when single treatments of 2-DG and diclofenac sodium were compared in both cell lines; thus, *p* < 0.0584 and *p* < 0.142 for MDA-241 and MCF7, respectively.

### 2.2. Cell Death Analysis by Apoptosis Assay

**Figure 2:**
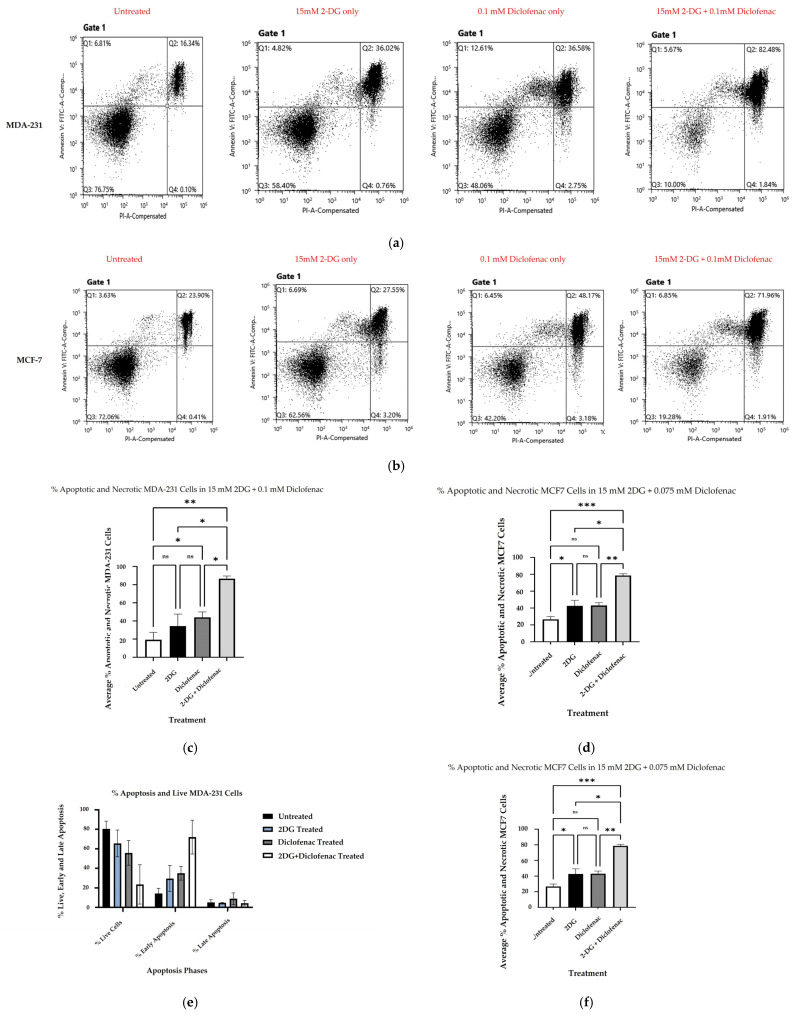
(Apoptosis assay) MDA-231 and MCF7 cells were separately plated and incubated overnight. The cells were then treated with therapeutic doses of 2DG and diclofenac in single and combinations treatments and incubated for 72 h before being stained with Annexin V and Propidium Iodide accordingly. Using a flow cytometer, percentages of live, early apoptotic and necrotic cells were determined in three independent experiments (*n* = 3) for each cell line. Results are presented in terms of mean percentages and ±SEM, with *p* ≤ 0.05 being considered statistically significant. As shown in dot plot diagrams (**a**,**b**), the combined treatment of cells generally increases the number of apoptotic cells than untreated and single treatments in both MDA-231 and MCF7, respectively. (**c**,**d**) show plots of the mean percentage of dead cells (apoptotic and necrotic cells) for both MDA-231 and MCF7, cell respectively, in different treatments. (**e**,**f**) show graph plots of mean percentages of live, early apoptosis cells and late apoptotic (necrotic) cells for MDA-231 and MCF7, respectively, in different treatment groups. ns indicates no significant differences between the groups. Significantly different groups, * = *p* < 0.05, ** = *p* < 0.01, *** = *p* < 0.001, respectively.

The cell death mechanisms were determined by Annexin V/Propidium Iodide staining and flow cytometry following the treatment of cell cultures with single and combination doses of 2-DG and diclofenac sodium. The mean percentages of dead cells (combined necrotic and apoptotic cells) of three independent experiments (*n* = 3) were evaluated against the percentage of live cells and compared. Results of multiple comparisons of mean percentages of dead cells revealed that the combination treatment of MDA-231 cells with 2-DG and diclofenac sodium significantly increased the percentage of dead cells (*p* < 0.003) when compared with untreated cells and (*p* < 0.004) in comparison with both single treatments (Figure 2a). Similarly, there was a glaring significant increase in the percentage of dead MCF7 cells in combination treatments (*p* < 0.0006) and (*p* < 0.004) when compared with both untreated and single treatments, respectively (Figure 2b). Results further revealed that although there was more cell death in diclofenac sodium treatment than in 2-DG treatments, the increase did not reach statistically significant levels in single treatments in either of the cell lines. Analysis of cells in both phases of cell death, however, showed that, in combination treatments, the percentage of cells in early apoptosis was, overall, highest in both cell lines, followed by diclofenac and 2-DG, respectively, while the single diclofenac treatment caused the highest number of cells to undergo (late apoptosis) in both cell lines (Figure 2c,d). Overall, the percentages of cells in late apoptosis were lowest compared with early apoptosis and live cells.

### 2.3. Determination of Reactive Oxygen Species

**Figure 3:**
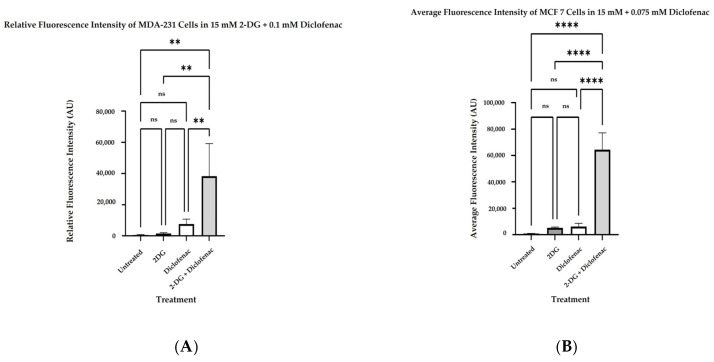
(Reactive oxygen species determination) MDA-231 and MCF7 cells were separately plated for overnight incubation and treated with GI50 doses of both single 2-DG and diclofenac sodium, as well as their combinations for 72 h. Cells were then stained with DHR-1232 and incubated for 2 h in the dark. Fluorescence intensities of five (*n* = 5) independent experiments were then obtained at 490/570 excitation and emission wavelengths for each cell line. Results of single and combination treatments were normalized with the blank (no cells) and compared with the controls (untreated samples). Results were presented as mean ± SEM with *p* ≤ 0.05 considered significant. ns indicates no significant differences between the groups. (**A**,**B**) show increase in ROS production as a function of relative fluorescence intensities (AU) in combination-treated samples compared with all other groups for both MDA-231 and MCF7 cells, respectively. Significantly different groups, ** = *p* < 0.01, **** = *p* < 0.00001, respectively.

The generation of reactive oxygen species in single and combination treatments of BCs with 2-DG and diclofenac sodium was evaluated by measuring the fluorescence intensity of DHR123 following cell staining. Five independent experiments (*n* = 5) were carried out and normalized with the blank (background), and their mean fluorescence intensities were compared with that of the control (untreated samples). Generally, all single and combined treatments showed a significant increase in ROS production when compared with the untreated samples. Multiple comparisons of mean fluorescence intensities showed that the combination treatment of both MDA-231 and MCF7 cells with 2-DG and diclofenac sodium significantly increases ROS generation when compared with untreated samples (*p* ≤ 0.001). Further, in both MDA-231 and MCF7 cells, there were significant increases in ROS production when combination treatments were compared with single treatments of 2-DG only (*p* ≤ 0.003) and diclofenac sodium only (*p* ≤ 0.031) in part for MDA-231 cells (Figure 3A) and in 2-DG only (*p* ≤ 0.022) and in diclofenac sodium only (*p* ≤ 0.018) for MCF7 cells (Figure 3B). Although there was some observable increase in ROS levels when the diclofenac sodium treatment was compared with 2-DG-treated samples, the increase was not statistically significant in both cell lines.

### 2.4. Wound Healing

**Figure 4:**
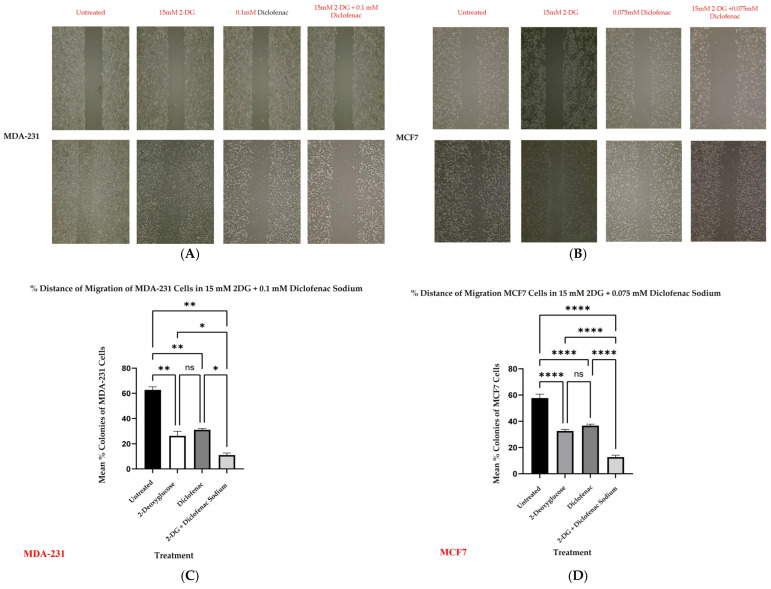
(**Wound healing assay**) scratch assay was performed following the treatment of BC cells (MCF7 and MDA). Cells were plated for overnight incubation and washed, and a scratch (wound) was created on the plates. Plates were rinsed and then separately treated with both single and combinations of 2-DG and diclofenac sodium at GI50 concentrations. Images of the wound were recorded at the beginning of the treatment, and the distances between the wound edges were measured at a 24 h interval, up to 72 h. Percentages of migrations were determined accordingly. The results of single and combination treatment samples of each cell line were compared with that of untreated cells. (**A**,**B**) show MDA-231 and MCF7 cells, both at zero time and corresponding after 72 h of treatment and treatment with either 2-DG only, diclofenac only, or combinations. (**C**,**D**) show the % distances of migration in different groups of untreated, 2-DG, diclofenac sodium, and combination treatments for both MDA-231 and MCF cells, respectively. Figures are represented in terms of mean and ±SEM; *p* ≤ 0.05 is considered significant. ns indicates no significant differences between the groups. Significantly different groups, * = *p* < 0.05, ** = *p* < 0.01, **** = *p* < 0.00001, respectively.

Used to mimic the in vivo metastatic ability of cancers including BCs, a migration assay was performed in MDA-231 and MCF7 cell cultures by separately creating scratches (wounds) in the adherent cells before single and combination treatments with the test compounds. The results of single and combination treatment samples of each cell line were compared with those of untreated cells. Figure 4A,B represent MDA-231 and MCF7 cells, both at zero time and corresponding after 72 h of treatment, with either 2-DG only, diclofenac only, or combinations. The percentage migrations in treated samples were compared with the percentages of untreated samples after 72 h. Figure 4C,D indicate comparisons across groups. Results of multiple comparisons of mean percentage migrations after 72 h incubation of untreated, 2-DG only, and diclofenac sodium only groups of both MDA-231 and MCF7 cells indicated that there was a significant increase in migration (reduction of wound size) across all the untreated and single treatment groups (*p* < 0.001) in both cell lines. However, when combination treatments after 72 h were compared with the initial wound sizes at zero hours, results showed that there was no significant reduction in wound sizes (no migration) in both MDA-231 (*p* < 0.08) and MCF7 (*p* < 0.196). A further comparison of mean percentages of untreated cell groups with single and combination treatments showed that there was an overall significant increase in the migration of untreated cells (*p* < 0.001) compared with all the treated MDA-231 cell samples. In MCF7 cells, however, results showed that there was a significant increase in migration among untreated samples compared with diclofenac sodium only and combination treatments (*p* < 0.002; *p* < 0.001), respectively. Additionally, when single treatments of 2-DG and diclofenac were compared with each other in MDA-231, results indicated that although there was an observable increase in migration in 2-DG-treated cells compared with that in diclofenac sodium-treated ones, the reduction in wound sizes was not statistically different in both groups. However, in MCF7 cells, there was a significant increase in migration of cells in 2-DG-only-treated cells (*p* < 0.043), but the diclofenac sodium-only treatment significantly inhibited cell migration (*p* < 0.196). Finally, although some observable migrations were noted when combined treatments were compared with both untreated, 2-DG-only, and diclofenac sodium-only treatments, there was generally no significant reduction in wound sizes in combination treatments of both MDA-231 and MCF7 cells as opposed to single treatments.

### 2.5. Immunoblotting

**Figure 5:**
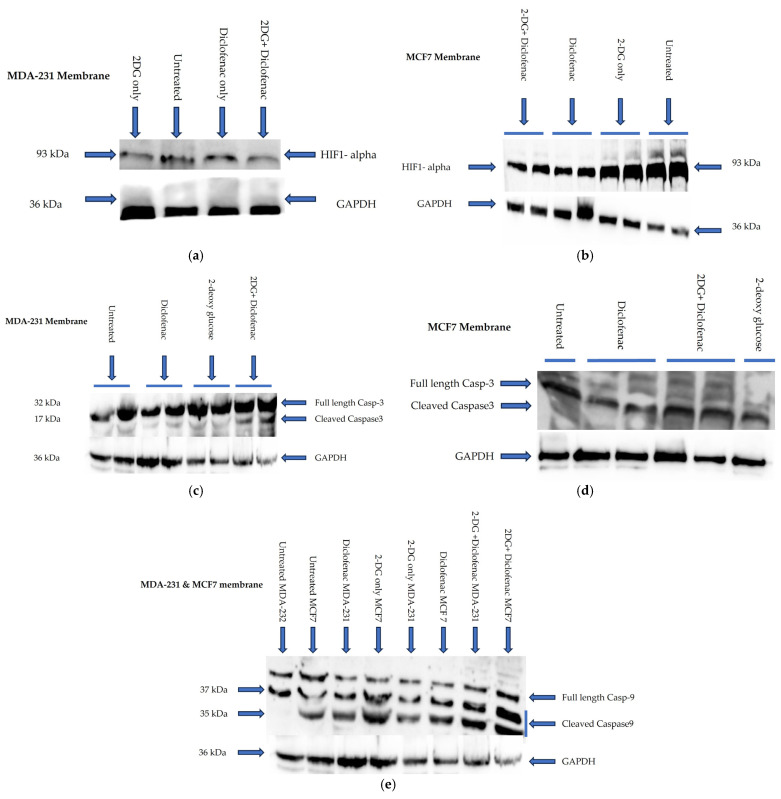
(Western blotting) MDA-231 and MCF7 cells were separately treated with 2-DG and diclofenac sodium in both single and combination forms at GI50 doses for 72 h. Proteins were extracted for Western blotting with specific antibodies of HIF1-alpha, Casp-3, and Casp-9. GAPDH antibody was used as an internal control. Untreated, single-treated, and combination treatment samples of both cell lines were separately loaded in different wells before gel resolutions. Representative results of three independent experiments (*n* = 3) are shown. (**a**,**b**) show immune blots of HIF1-alpha expression in MDA-231 and MCF7 cells, respectively, while (**c**,**d**) represent cleavages of immunoblots of Casp-3 in MDA-231 and MCF7 cells, respectively. Additionally, (**e**) shows cleavages of Casp-9 in both MDA-231 and MCF7 cells.

To further examine the synergistic effects of 2-DG and diclofenac sodium on the apoptosis and metastasis of breast cancer cells, Western blotting was performed to examine the effects of a combined treatment of the drugs on the expression of HIF-1α and the cleavage of pro-apoptotic proteins, Caspase-3 (Cas-3) and Caspase-9 (Cas-9). MDA-231 and MCF7 cells were separately plated for overnight incubation and later treated with single and combination doses of 2-DG and diclofenac sodium both at IG50 concentration for 72 h. Cells were then lysed, and proteins were extracted for Western blotting. Anti-HIF-1α, anti-Casp-3, and anti-Casp-9 were used as primary antibodies. Additionally, anti-GAPDH was used as internal control in both cases. Untreated, single-treatment, and combination-treatment samples were loaded in separate wells for both cell lines. Results showed that combination treatments with 2-DG and diclofenac sodium in both cell lines separately suppress the expression of HIF-1 alpha in both MDA-231 (Figure 5a) and MCF7 (Figure 5b) compared with untreated and single treatments. In addition, it was also observed that combination treatments of both cell lines with the drugs enhanced the cleavages of Caspase-3 (Figure 5c,d) in MDA-231 and MCF7 cells, respectively, as well as Caspase-9 (Figure 5e) for both cell lines, compared with all other groups.

### 2.6. Colony Formation

**Figure 6:**
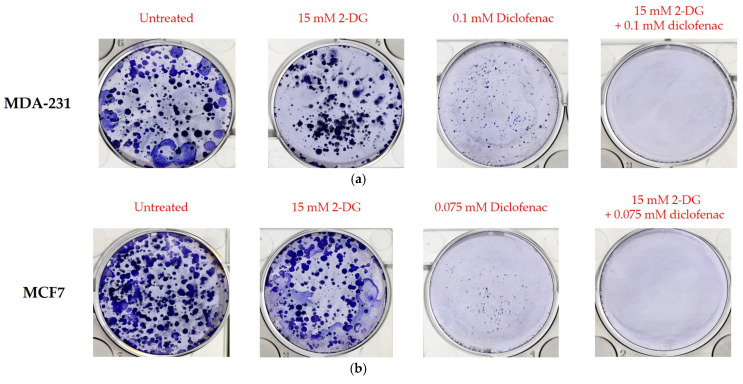

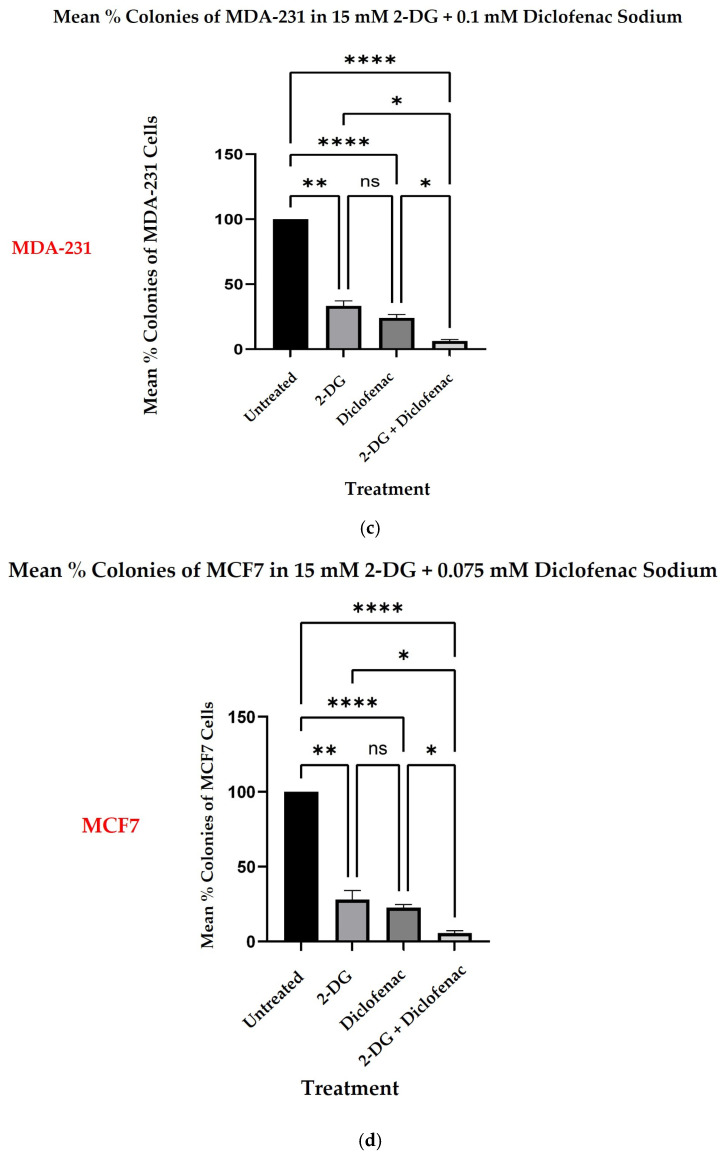
(Colony formation assay) MDA-231 and MCF7 cells were separately plated overnight and treated with single and combination doses of 2-DG and diclofenac sodium for 14 days. Cells were then later stained with Coomassie blue. (**a**,**b**) represent colonies of MDA-231 and MCF7, respectively, for three independent experiments (*n* = 3) in different treatments. (**c**,**d**) show mean ± SEM of MDA-231 and MCF7, respectively, with *p* < 0.05 considered significant for MDA-231 and MCF7 cells. ns indicates no significant differences between the groups. Results show that a combined treatment of both cell lines with 2-DG and diclofenac significantly inhibits colony formation. Significantly different groups, * = *p* < 0.05, ** = *p* < 0.01, **** = *p* < 0.00001, respectively.

Colony formation or clonogenic assay was carried out to evaluate the effects of single and combination treatments of 2-DG and diclofenac sodium on the expansion of MDA-231 and MCF7 cells. Cells were seeded and treated for 14 days prior to staining with Coomassie blue. Colony sizes > 0.5 mm of three independent experiments (*n* = 3) were then counted and expressed as percentages of the baseline (untreated controls). Figure 6a,b represent colonies of MDA-231 and MCF7 cells in different treatments respectively. Results (Figure 6c,d) for MDA-231 and MCF7, respectively indicated that there was an overall significant reduction in colony numbers among all treated groups (*p* < 0.001) compared with untreated samples in both cell lines. Additionally, it was noted that a single treatment of cells with diclofenac sodium, on one hand, and combination treatment, on the other hand, caused a significant reduction in colonies (*p* < 0.001 and *p* < 0.016), respectively, compared with 2-DG-only treatment of MDA-231 cells. However, while the combination treatment significantly reduced the colony numbers in MCF7 cells compared with 2-DG-only treatment (*p* < 0.007), there was no significant change in the number of colonies (*p* < 0.082) when 2-DG and diclofenac-only treatments were compared. Generally, results indicated that the combination treatment significantly caused more reduction in colony numbers in both MDA-231 and MCF7 cells than in all the single treatments. The reduction in colonies in MCF7 following combination and diclofenac sodium-only treatments was seen to be so extensive to the extent that the difference in their effects on the inhibition of colony formation could not be discerned when the two treatment groups were compared. However, the combination treatment of MCF7 cells also showed a significant reduction in colony numbers compared with both single treatments.

## 3. Discussion

The in vitro anti-cancer effects of drugs on solid tumors can be evaluated by various cell-based assays and methods, such as analysis of cell viability by 3-(4,5-dimethylthiazole-2-yl)2,3diphenyl tetrazolium bromide (MTT assay), which is based on the principle that the mitochondrial oxidoreductase found in viable cells in cell cultures converts the MTT reagent into a purple soluble formazan product, which can be quantified spectrophotometrically, at a 570 nM wavelength [34,35]. Accordingly, in agreement with the literature [7,9,18], both 2-DG and diclofenac sodium separately exhibited anti-cancer effects in MD-231 and MCF7 cells in a linear dependent manner when evaluated in cell-based MTT viability assay (Figure 1a,b). The anti-cancer effects were markedly increased in combination treatments compared with single treatments in both cell lines (Figure 1b,c). This showed that there were glaring additive effects of both drugs when used in combination against the cells. Despite multiple studies revealing that TNBC (MDA-231) cells are more resistant to chemotherapy than non-TNBC (MCF7) cells [4,5,36], we did not find any difference in anti-cancer effects between the two cell lines, in either single and combination treatment. The exact signal mechanisms leading to cytotoxicity were not immediately established, and probably need to be explored. Additionally, apoptosis assays can be performed by the use of an Annexin V/Propidium Iodide assay and evaluated by flow cytometry. During the early phases of cellular apoptosis, phosphatidylserine, which is usually localized on the inner plasma membrane of cells, becomes exposed to the surface, and Annexin V can bind to it, while the lipid-soluble propidium iodide binds to the nucleus of a disintegrated cell in late apoptosis (necrotic stage) [37,38,39]. The fluorescence intensities of the two stains can then be quantified and analyzed by flow cytometry. Apoptotic mechanisms are regulated by cysteine-aspartate proteases called caspases. Originally, caspases exist in cells as inactive zymogens, which become active only after proteolytic cleavage [40,41]. Caspases are largely classified into two groups: initiator caspases (Caspases 2, 8, 9, and 10) and effector caspases (Caspases 3, 6, and 7) [42,43,44]. Initiator caspases are up-regulated in early apoptotic signals, while the effector caspases are activated by initiator caspases [41]. Therefore, we found out that a combination treatment of MDA-231 and MFC7 cells with 2-DG and diclofenac sodium increased apoptosis in both cell lines, compared with single treatments, when evaluated by Annexin V/Propidium Iodide assay (Figure 2a–d), with more cells seen in early apoptosis than late apoptosis (Figure 2e,f). As expected, immunoblotting also indicated marked cleavage of initiator Caspase-9 and effector Caspase-3 in both cell lines (Figure 5c–e). Consistent with the previous findings [7,9], the results of both Annexin V/PI assay and immunoblotting overall indicated substantial additive effects of the drugs on the activation of apoptosis and necrosis of both MDA and MCF7 cells with subtle variations between the cell lines, hence depicting the synergy of the drugs in the apoptotic cell death processes. Further, increasing evidence shows that the enhanced generation of intracellular reactive oxygen species (ROS) in tumor cells is cytotoxic [18,44]. As such, the effects of exogenous molecules such as 2-DG and diclofenac sodium that would potentially lead to the intracellular generation of ROS need to be explored. Produced during the metabolic course, ROS in neoplastic cells have a dual purpose depending on the concentration [45]: On one hand, lower- and medium-level concentrations of ROS [46] induce mitogen-activated protein kinase/extracellular signal-regulated Kinase 1 or 2 (MAPK/ERK1/2) andPI3K/Akt, which are largely cell survival signaling cascades. ROS also activate the nuclear factor Kappa-light chain enhancer of activated B-cells (NF-kB), matrix-associated metalloproteinases, and vascular endothelial growth factor receptor (VEGF), among other pathways that enhance tumor metastasis and migration [45,47]. On the other hand, elevated concentration of ROS in tumor cells induces apoptotic-mediated tumor cell killing, mainly by interfacing with the mitochondrial transition permeability pore (mPTP), hence effectively interrupting the oxidative phosphorylation process. This leads to cytochrome C (cyt C) leakage to the cytosol, which eventually forms apoptosome in combination with apoptotic peptidase activating factor 1 (Apaf-1), which combines and cleaves pro-Caspase-9 to its active form Cse-9. The activated Caspase-9 then activates the apoptotic effector Caspase-3, which induces apoptosis [48,49]. Accordingly, our findings showed that the generation of ROS was higher in combination treatments as opposed to single and untreated ones (Figure 3A,B), with no difference in ROS generation between the cell lines, indicating that 2-DG and diclofenac sodium have the additive effect in enhancing metabolic pressure in the cells [50,51]. Although not specifically determined as the main cytotoxic mechanism, the increase in ROS generation in combination treatment undoubtedly underscores the collective cytotoxic effects of these drugs in the cells, as espoused in previous findings [45]. On the other hand, metastatic neoplastic BCs form colonies while at the same time undergoing hematogenous spread through lymphatic and blood vessels, making them invasive of other body organs and establishment as they dissolve the underlying basement membrane [52]. Like other tumor survival mechanisms, metastasis is a tightly regulated process, involving many regulatory elements and mechanisms. Mimicking in vivo mechanisms, in vitro wound healing (migration assay) can be performed to evaluate the metastatic ability of cells. This can be validated by evaluating the expression of HIF-1α. Due to underlying hypoxic conditions in solid tumors, the role of hypoxia-inducible factors (HIFs) cannot be over-emphasized. Mechanistically, HIFs regulate various signaling cascades geared towards adapting the cancer cells to hypoxic conditions in order to allow invasiveness and metastasis. These include PI3K/Akt/MTOR axis, NOX. and Wnt/*B*-catenin pathways. Other pathways regulated by HIFs are cytoskeleton protein expression, epithelial mesenchymal transition, and angiogenesis [11,53]. While several HIF subtypes participate in hypoxic regulation in different ways, the function of HIF-1α, as a key mediator of angiogenesis, has been widely studied and used as a target molecule for cancer therapies [54]. HIF-1α regulates the expression of angiogenic molecules, including stromal-derived factor 1 (SDF1), vascular endothelial growth factor and its binding receptor (VEGF/VEGFR), placental growth factor (PGF), platelet-derived growth factor B (PDGFB), and regulation of both angiopoietin 1 and 2 (ANGPT 1 and 2) [55]. In the same breath, therefore, the inhibition of the migration of MDA-231 and MCF7 cells was evaluated and found apparent in combination treatments of 2-DG and diclofenac sodium compared with single-treated and untreated groups (Figure 4A–D), with reduced expression of HIF-1α (Figure 5a,b). As expected, and in agreement with previous findings, TNBC (MDA-231) cells, which are widely regarded as more invasive and resistant to therapies [4], exhibited some subtle expression of HIF-1α even in combination treatments, unlike their non-TNBC (MCF7) counterparts, which are known to be more sensitive to therapies and moderately invasive. Consistent with previous studies, nevertheless [7,9,54,55], these findings collectively indicated that combination treatments of 2-DG and diclofenac sodium would substantially inhibit HIF-1α and its mediated signals for angiogenesis and quiescence, hence inhibiting overall in vivo metastasis, in both MDA-231 and MCF7 cell lines, thus inhibiting invasion to distant body cells in both BC cell lines. Finally, results also indicated that the combination treatment of the cells substantially inhibited aggressiveness by reducing the colony formation ability, and even though TNBC (MDA-231) cells are known to be more aggressive than their counterparts, non-TNBC (MCF7) cells [4,7,56], there was limited variation in the extent of colony formation between the two cell lines when compared (Figure 6a–d). The limitation of colony formation underscored the additive effect of 2-DG and diclofenac in the overall cytotoxicity of both cell lines, which was consistent with the viability findings.

## 4. Conclusions

Although this study was limited in scope to in vitro experiments to generally determine anti-cancer effects of these compounds in BCs due to inadequate funding—and hence, no in vivo experiments were involved—these findings provide experimental evidence of the anti-cancer synergy of 2-DG and diclofenac sodium in breast cancer cells. The additive anti-cancer effects of these compounds on the two cell lines of BCs (MDA-231 and MCF7) do not, however, differ substantially, despite the fact that MDA-231 is known to be more resistant to other therapies than their counterparts, MCF7. This study does not show the exact molecular mechanisms of actions of the drugs on the cells. On this basis, we recommend future studies to further evaluate and pinpoint the exact signaling or other molecular pathways and mechanisms of actions of these combination treatments in breast cancer cells and in animal experiments so as to further elucidate their anti-cancer effects in a much broader context. In addition, this study clearly compared the effects of combination treatments of 2-DG and diclofenac sodium in cells, and assumes that each drug acted independently of each other on cells. It does not delve into the details of the exact fractional effects of each drug in the mixture on the cells, taking into account potential drug interactions. Therefore, we recommend further evaluation of specific doses for combination treatments, potential interactions between them, and the exact fractional effects of each one of them on BCs. The glycolytic targeting of the cells with these drugs is generally known. Since this study has revealed the overall anti-cancer and cytotoxic potential of these molecules on BCs, and considering the high metabolic rate of tumors in general, these results would inform future studies on tumor metabolism at various levels of cellular metabolism. These include, but are not limited to, studies aimed at elucidating the drug effects on enzymes and genes of the pyruvate dehydrogenase complex, TCA cycle, oxidative phosphorylation, studies on mitochondrial bioenergetics such as extracellular acidification, oxygen consumption, and mitochondrial potential. Finally, the present results have also revealed that the combined treatment of the cells limits the expression of HIF1α, a key mediator of metastasis. This molecule works in cohort with other key mediators of angiogenesis, such as VEGF, TGFα, TGFβ, FGF, matrix metalloproteinases, interlukin-1 (IL-1), and TNFα; future studies would therefore be recommended to investigate the effects of combined treatments of cells with the drugs on the expressions of these angiogenic molecules in cancer therapy.

## 5. Materials and Methods

### 5.1. Cell Cultures

Breast cancer cell lines: MDA-231 and MCF7 purchased from the American Type Culture Collection (Manassas, VA, USA) were routinely propagated in a humid 5% CO_2_ atmosphere at 37 °C in DMEM low glucose and RPMI growth media (Biosera, Nauaille, France), respectively. Both media were supplemented with 10% of **fetal bovine serum** (Thermo Fisher, Life Technologies, Milan, Italy). Both 2-DG and diclofenac sodium salt were obtained from Sigma Aldrich (Budapest, Hungary).

### 5.2. 3-(4,5-Dimethylthiazol-2-Yl)-2,5-Diphenyltetrazolium Bromide (MTT) Assay

Following treatment with the test drugs, cell viability was evaluated by cell survival assay (MTT) in line with the protocol [57], with appropriate modifications. Preliminary experiments were separately performed to determine the concentrations of 2-DG and diclofenac sodium that would cause approximately 50% of cell death (GI50). Next, 1 × 10^4^ cell density/well of both MDA-231 and MCF7 was seeded in 96-well cell culture plates for overnight incubation prior to treatment. The cell cultures were then treated with GI50 doses of 2-DG and diclofenac sodium separately and in their combinations for 72 h. Consequently, the previous culture media were replaced with 100 µL/well fresh media containing 10% MTT substrate. This was followed by 2 h incubation in the dark at a 37 °C temperature. Subsequently, the media (with MTT substrate) were discarded, and 100 µL/well dimethyl sulfoxide (DMSO) was added to develop the soluble purple formazan product. Finally, the optical density (OD) of the formazan product was measured using the Glomax^®^-multi-instrument (Promega, Madison WI, USA) at an absorbance wavelength of 570 nm. The experiments were repeated three times, with each one of them running in at least three parallels.

### 5.3. Reactive Oxygen Species (ROS) Determination

The production of ROS in cell cultures was examined by measuring ROS concentration following the guidelines in the protocol [47]. Briefly, 1 × 10^4^ cell density/well of both MDA-231 and MCF7 cells was seeded on 96-well cell culture plates and incubated overnight prior to treatment. The cells were then washed with phosphate-buffered saline (PBS, Biowest, Neuville, France) and treated with GI50 doses of 2-DG, diclofenac sodium, and their combinations for 72 h. Afterwards, 2 µM Dihydrorhodamine 123 (DHR123) fluorescent dye was added at 100 µL/well, and the cell cultures were incubated in darkness for 2 h. Finally, at an excitation wavelength of 490 nm and an emission wavelength of 510 nm–570 nm, the fluorescence intensity of DHR123 was evaluated by the use of the Glomax^®^ multi-instrument (Promega, Madison, WI, USA). For every treatment, each experiment was performed in triplicate and independently repeated three times.

### 5.4. Colony Formation Assay

Cell survival was assessed by colony formation or clonogenic assay, following the guidelines [58]. In brief, 3 × 10^3^ of cell density/well of MDA-231 and MCF7 cells/well single suspensions was plated in DMEM low glucose and RPMI media, then incubated overnight in 6-well plates, and later treated with IG50 doses of 2-DG, diclofenac, and their combinations. The cell cultures were then incubated further for 14 days. Finally, the cultures were washed with PBS (Biowest, Neuville, France) and stained with 0.1% Coomassie brilliant blue, R250 (Merk, Darmstadt, Germany), mixed with 30% methanol and 10% acetic acid solutions. After rounds of washing, the plates were dried, and the colonies were counted using a phase contrast microscope The images were also scanned accordingly. The experiments were separately repeated three times.

### 5.5. Wound Healing Assay

Cell migration was assessed by wound healing assay guidelines [59]. Shortly, 3 × 10^5^ cell density/well of MDA-231 and MCF7 cells was seeded on the 6-well culture plates in DMEM low glucose and RPMI media, respectively, supplemented with 10% *v*/*v* FBS and incubated in 37 °C and 5% CO_2_ atmosphere for 24 h. Following confluency of between 80% and100%, the cell cultures were retrieved, media-aspirated, and washed with PBS. Next, using a sterile 200 µL plastic pipette tip, a perpendicular scratch (wound) was physically created on each plate well. Afterwards, the culture plates were thoroughly rinsed with PBS and culture media. The medium was then aspirated and replaced with low serum media (2% FBS) containing GI50 doses of 2-DG, diclofenac sodium, and their combinations. Images of the scratch were then taken immediately following treatment (at zero time), using phase contrast microscopy. The initial sizes of the wounds in five independent experiments (*n* = 5) were measured in terms of distances between each edge of the scratches and the other in all treatment groups of both cell lines. The distances were measured at the beginning (zero hour) and in intervals of 24 h up to 72 h. The final distances were subtracted from the initial distances, and results were expressed in percentages of the initial distances to show the extent of cell migration toward the wound areas. Each assay was repeated three times.

### 5.6. Apoptosis Assay

With strict adherence to the manufacturer’s guidelines, cell death mechanisms were assessed by evaluating live, early apoptosis, and necrotic phases cell cultures using an Annexin V/Propidium Iodide assay kit (Luminex Corporation, Austine, TX, USA). Initially, cells were prepared following the apoptosis assay protocol [40], with slight modifications prior to staining. Then, 3 × 10^5^ cell disunity/well of both MDA-231 and MCF7 cells was seeded in 6-well cell culture plates in DMEM low glucose and RPMI media, respectively, both supplemented with 10% *v*/*v* FBS. The cells were then treated with GI50 doses of 2-DG, diclofenac sodium, and their combinations for 72 h. Cells were subsequently collected at 4 °C and counted, and a dilution of 5 × 10^5^/mL was made, before washing appropriately in a cell staining buffer. Next, 5 µL Annexin V FITC and 10 µL Propidium Iodide reagents were added to every sample. Following 15 min of incubation at room temperature in the dark, a 300 µL Annexin V binding buffer was added to each sample before measurement using a MUSE cell analyzer instrument. The Annexin V-FITC positivity was recorded as either early apoptotic or late apoptotic phases, while the Annexin-FITC negative events (PI positive) were considered to be in the necrotic phase. Each experiment was performed in duplicate, and three independent experiments were carried out separately.

### 5.7. Western Blotting

The expression of a pro-metastatic protein, hypoxia-inducible factor-1 alpha (HIF-1 alpha), and cleavage of apoptotic proteins, Caspase-3 and Caspase-9, was assessed by Western blotting following the guidelines [12]. To begin with, MDA-231 and MCF7 cells were plated on 10 cm cell culture plates and incubated overnight to achieve between 80% and 100% confluency. The cells were then treated with the GI50 doses of 2-DG diclofenac sodium and their combinations for 72 h. The cells were subsequently washed with PBS, harvested, and lysed by a cold 4 °C lysis buffer on ice at PH 7, containing 20 mM HEPES, 10 mM EGTA, 2 mM EDTA, and 1 M NaOH and Na_3_VO_4_ protease inhibitor. By scraping, proteins were then extracted from the cells with calibrated sonication, cooling, and heating processes. Protein concentration and normalization were performed by the modified Bradford method, according to the manufacturer’s instructions, before diluting with a 4x protein sample buffer (SB). Approximately 50 µg of each protein sample, together with the protein ladder, was then loaded into the prepared 12% polyacrylamide gels, containing sodium dodecyl sulphate (SDS), before resolving the gels accordingly, for 1 h 10 min, to allow protein separation. The gels were then electroblotted for a further 1 h onto a polyvinylidene fluoride (PVDF) membrane, stained with Ponceau S for 15 min, and allowed to dry. The membranes were then cut according to the molecular weight of the desired protein, rinsed with Tris-Buffered Saline with Tween-20 (TBST), and blocked with 3% non-fat casein for 1.5 h at room temperature, before being separately exposed to respective primary antibodies diluted in blocking buffer at 4 °C. Following rounds of washing with TBST, Horseradish peroxidase-conjugated secondary antibodies anti-mouse IgG and anti-rabbit IgG from Sigma Aldrich (Budapest, Hungary) were added to the membranes at 1:5000 dilutions, before incubating further for 1.5 h at room temperature. Finally, an enhanced chemiluminescence detection system (Amersham Bioscience, Piscataway, NJ, USA) was used to visualize the band films, before scanning and evaluating their pixel volumes, using NIH ImageJ software (version 4.1, Bethesda, MD, USA). All experiments were repeated three times independently.

### 5.8. Data Analysis

Data were expressed as mean ± SEM. Concentration-dependent effects of 2-DG and diclofenac sodium on cell viability were tested by the simple linear regression model. Multiple comparisons of means between groups of untreated (vehicle control), single-treatment, and combination treatments were performed by one-way ANOVA with the Tukey post hoc test. Differences were considered as significant with *p* ≤ 0.05. Data were analyzed by the use of IBM SPSS statistical analysis ver29.0 software (IBM cooperation, New York, NY, USA), and NIH-ImageJ software graphs were produced by GraphPad Prism software, version 10.4.

## Data Availability

All data generated or analyzed during this study are included in this published article.
